# Dual-arm admittance control using conformal geometric algebra

**DOI:** 10.3389/frobt.2026.1807613

**Published:** 2026-05-13

**Authors:** Tobias Löw, Mariana de Paula Assis Fonseca, Vitalii Pruks, Graham Deacon, Jelizaveta Konstantinova, Sylvain Calinon

**Affiliations:** 1 Idiap Research Institute and Ecole Polytechnique Fédérale de Lausanne (EPFL), Lausanne, Switzerland; 2 Ocado Technology, Hatfield, United Kingdom

**Keywords:** admittance control, conformal geometric algebra, dual-arm manipulation, impedance control, robotic manipulation

## Abstract

We propose a task-space admittance controller for dual-arm robotic systems using conformal geometric algebra. The controller is a reinterpretation of a previous work using dual quaternion algebra. By introducing conformal geometric algebra, we aim to enhance the geometric expressiveness, which simplifies the modeling of various tasks and opens doors to more complex applications, such as the modeling of multiple points of contact on the robotic arm in a whole-body manipulation task. We first show the derivation of the controller for a single-arm robot, which is then extended to a dual-arm robot. The closed-loop system is therefore composed of an outer loop admittance controller that imposes the apparent impedance, and an inner loop that transforms the twist acceleration to a control input that is sent to the robot. Experiments executed on a setup with two LBR KUKA iiwa 14 R820 robots with a force/torque sensor in each end-effector show good performance of the proposed controller for both single and dual-arm tasks. Namely, the system was able to reach the desired poses in the absence of external wrenches, while moving in a compliant manner in the presence of external wrenches, adapting the robot’s motion to keep the desired impedance.

## 1 Introduction

Nowadays, robots can be found in a variety of environments, from the factory floor to the home. The various different tasks that robots are then expected to execute in these environments increasingly require the robots to interact with objects in a way that goes beyond traditional parallel jaw grippers or suction cups that are often featured on single arm manipulators. Many applications, such as depalletizing large and deformable items, may call for dual-arm manipulators for picking and placing. For those systems, the coordination between two arms is vital when grasping, lifting, and transport objects of varying shapes and fragility.

A fundamental challenge in robotic manipulation lies in achieving safe and compliant physical interaction. Robots inevitably experience external wrenches (forces and torques) during contact with objects and the environment. Unmanaged, these can lead to damage or instability. Impedance and admittance control define a desired dynamic relationship between the wrenches applied to the robot and its resulting motion, allowing robots to react compliantly by behaving like a virtual spring-damper-mass system.

When working with impedance or admittance controllers, it is important to define a stiffness term that is consistent with the task geometry, for both position and orientation terms, to prevent unnatural behavior. Considering that, Caccavale et al. proposed to use an energy-based impedance equation, and to use the imaginary part of the unit quaternion to represent the orientation displacement between the desired and current frames Caccavale et al. (1999). However, in this work, the authors propose a stiffness that is geometrically consistent only for infinitesimal displacements. Therefore, they extended the previous work to have a stiffness that is geometrically consistent for also finite displacements Caccavale et al. (2008). A reformulation of this controller using dual quaternion algebra showed an exponential decay of the error norm due to the linearity of the stiffness term, while also not suffering from the problem of topological obstruction Fonseca et al. (2020).

Coordinating the motion of a dual-arm manipulator requires control strategies that enable collaborative behavior. To this end, the cooperative dual-task space (CDTS) was proposed Adorno et al. (2010). This approach unifies the end-effector poses into an absolute and a relative poses that are expressed using dual quaternions. The CDTS was then used to formulate an admittance controller for a dual-arm mobile robot Fonseca (2021), which was then further improved by also introducing an adaptive admittance behavior Nebbia Colomba et al. (2022). Using conformal geometric algebra (CGA), the CDTS was extended by introducing the cooperative pointpair primitive, and integrated into an optimal control formulation and use inverse dynamics control to compute desired torque commands Löw and Calinon (2024a). While this approach enabled a compliant behaviour of the manipulators, it did not take into account actively controlling desired interaction.

Conformal geometric algebra provides a powerful and unified mathematical framework for representing geometric entities (points, lines, planes, spheres, etc.) and their relationships. This intrinsic capability allows for easier modeling of complex tasks, enabling control strategies that focus, for instance, on interacting with surfaces or lines rather than just specific points, thereby relaxing task constraints.

### 1.1 Relation to geometric impedance control

Recent work on geometric impedance control (GIC) has formulated impedance and admittance controllers directly on SE(3) and its Lie algebra se(3) Seo et al. (2024a), Seo et al. (2024b). These approaches achieve geometrically consistent error definitions by working on the group and algebra of rigid body transformations, sharing the underlying motivation of our work. Compared to SE(3) -based formulations, the motor group/CGA approach offers several structural advantages. First, position and orientation are unified in a single bivector error $B_{e}=-\log\left({\tilde{M}}_{q}M_{d}\right)$ , whereas SE(3) formulations must handle the $R^{3}$ and SO(3) components separately. Second, and more distinctively, CGA directly embeds geometric primitives as algebraic elements in the same algebra, enabling the controller formulation of Section 3.5, which targets interaction with geometric primitives rather than poses. This capability has no counterpart in SE(3)-based controllers. Third, while SE(3)-based dual-arm controllers following Adorno et al. (2010) operate on poses, the CGA formulation of the CDTS introduced in Löw and Calinon (2024a) enables the cooperative pointpair primitive Equation 7, extending compliant dual-arm coordination to tasks defined by geometric primitives.

### 1.2 Statement of contributions

In this work, we are employing CGA to formulate an admittance control strategy building upon Fonseca et al. (2020) and show how it not only enables the integration of geometric primitives within the control formulation, but also allows for a natural extension to more than one manipulator. Our contributions therefore are:

- Formulation of an admittance control scheme in CGA.
- Extension of the proposed controller for a dual-arm robotic system involving parallel kinematic chains.
- Formulation of the controller for geometric primitives.
- Extension of unwinding solution for CGA.
- Experimental validation of the proposed controller on a single-arm and dual-arm setups with Kuka iiwa robots.

## 2 Mathematical background

### 2.1 Geometric algebra

Geometry is a fundamental part of robotics. Over the years, many frameworks have been used, such as standard vector analysis, matrix algebra, and quaternions. Geometric algebra (GA) has gained attention recently due to its property of unifying many concepts into a single algebra.

The GA is based on a multiplication operation called geometric product, which is a sum of an inner product and an outer product. This concept leads to the simplification of many complex equations. In robotics, these operations (geometric, inner, and outer products) allow translations and rotations to be treated in the same way, without requiring the switch between different algebras. Moreover, GA allows geometric operations to be computed very fast, with compact codes Löw et al. (2025).

In this article, we use the specific variant known as conformal geometric algebra (CGA) $G_{4,1}$ . The CGA extends the Euclidean space $R^{3}$ by two additional basis vectors, one of which squaring to +1 and the other to −1. The conformal model is then found by a change of basis that introduces the basis vectors $e_{0}=\frac{1}{2}\left(e_{5}-e_{4}\right)$ and $e_{\infty }=e_{4}+e_{5}$ , which can be understood as a point at the origin and one at infinity. We will use the following notation throughout the paper: x to denote scalars, x for vectors, X for matrices, X for multivectors and X for matrices of multivectors.

#### 2.1.1 Geometric primitives

One of the advantages of GA is the geometric significance of its elements. An object can directly represent geometric primitives, such as lines, spheres, and planes, as well as orthogonal transformations, such as rotations and translations. In general, geometric primitives can be constructed using the outer product, i.e.,

$$X=\overset{n}{\underset{i=1}{⋀}}P_{i},$$

where a different geometric primitives is computed depending on the number of points n . For example, three points form a circle or, if one of those points is at infinity, a line. The geometric primitives are essentially subspaces of the algebra and formally they define a nullspace under the outer product

$${\mathrm{NO}}_{G}\left(X\right)=\left\{\vec{x}\in R^{3}:\;P\left(\vec{x}\right)\wedge X=0\right\}.$$

This means that the set of conformalized Euclidean points that result in zero using the outer product forms the geometric primitives. We have shown previously how this outer product formulation can be formulated within an optimal control framework to define reaching tasks involving the geometric primitives Löw and Calinon (2023).

### 2.2 Rigid body transformations in CGA

For the purpose of robot kinematics and dynamics, we are interested in a representation of the special Euclidean group SE(3) , which is the group of rigid body transformations. It is well known that the group $Spin\left(3\right)⋉R^{3}$ is a double-cover of SE(3) , which can be represented as dual quaternions. In CGA, this group is represented as the group of *motors* $M=Spin\left(3\right)⋉R^{3}$ . The motor group M is a Lie group and we can find its Lie algebra as the bivector algebra $B_{M}=\left\{e_{23},e_{13},e_{12},e_{1\infty },e_{2\infty },e_{3\infty }\right\}$ . Elements of the Lie algebra $B_{M}\in B_{M}$ can be mapped to group elements M∈M via a surjective map called the exponential map $\exp:B_{M}\to M$ . Accordingly, its inverse operation for projecting group elements to the Lie algebra is named the logarithmic map $\log:M\to B_{M}$ . Choosing the canonical decomposition of motors M∈M as

M=TR,

where T is a translator and R is a rotor. Then using $B_{M}=B_{T}+B_{R}$ , the exponential maps can be found as

$$T=1-\frac{1}{2}B_{T},$$

where $B_{T}\in span\left\{e_{1\infty },e_{2\infty },e_{3\infty }\right\}$ , and

$$R=\cos\left(\frac{1}{2}\left\|B_{R}\right\|\right)-\sin\left(\frac{1}{2}\left\|B_{R}\right\|\right)\frac{B_{R}}{\left\|B_{R}\right\|},$$

where $B_{R}\in span\left\{e_{23},e_{13},e_{12}\right\}$ . Note that it is the bivector subalgebra $B_{M}$ that plays the role of the Lie algebra of the motor group M , not CGA as a whole.

Motors can be used to transform any multivector within the algebra, i.e., they can be used to transform geometric primitives. This adjoint operation is formulated as the product

$$X^{\prime }=MX\tilde{M},$$

where $\tilde{M}$ denotes the reverse of the motor M , which for motors is equivalent to their inverse, i.e., $\tilde{M}=M^{-1}$ , since by the group constraint they have unit norm $M\tilde{M}=1$ .

By taking the derivative of the logarithmic map w.r.t. time we can derive the relationship between the time derivatives of motors and bivectors

$$\dot{B}=\frac{\partial }{\partial t}B=\frac{\partial }{\partial t}\log\left(M\right)=M^{-1}\frac{\partial }{\partial t}M=\tilde{M}\dot{M}.$$

### 2.3 Robot modeling using CGA

The forward kinematics given the current joint configuration q of serial kinematic chains can be found via the product of joint motors, i.e.,

$$M\left(q\right)=\overset{n}{\underset{j=1}{\prod }}M_{j}\left(q_{j}\right)=\overset{n}{\underset{j=1}{\prod }}\exp\left(q_{j}B_{j}\right),$$ (1)

where $B_{j}$ is the bivector describing the screw axis of the j -th joint. For brevity of the notation we will be using $M_{q}$ instead of M(q) for the end-effector motor at configuration q in the rest of the article. We described the derivation of the analytic $J_{q}^{A}$ and geometric $J_{q}^{G}$ Jacobians in Löw and Calinon (2023). Expressed w.r.t. to the end-effector motor their relationship can be found as

$$J_{q}^{G}=-2{\tilde{M}}_{q}J_{q}^{A}.$$ (2)

Since the analytic Jacobian can be used to derive the time derivative of the end-effector motor

$${\dot{M}}_{q}=J_{q}^{A}\dot{q},$$

and the geometric Jacobian for finding the end-effector twist $V_{q,\dot{q}}$

$$V_{q,\dot{q}}=J_{q}^{G}\dot{q},$$ (3)

it is straightforward to relate the bivector time derivative of the end-effector motor ${\dot{B}}_{q}$ to the end-effector twist $V_{q,\dot{q}}$

$$V_{q,\dot{q}}=-2{\dot{B}}_{q}=-2{\tilde{M}}_{q}{\dot{M}}_{q}.$$ (4)

Mathematically, the space of twists is equivalent to the space of bivectors $B_{M}$ . The wrenches W are dual to twists, and they can be found explicitly in CGA using multiplication by the conjugate pseudoscalar, i.e., $W=VI_{c}$ , where $I_{c}=Ie_{0}$ Hestenes (2010) and $I=e_{\mathrm{0123\infty}}$ is the standard pseudoscalar of CGA.

### 2.4 Cooperative dual task space

**FIGURE 1:**
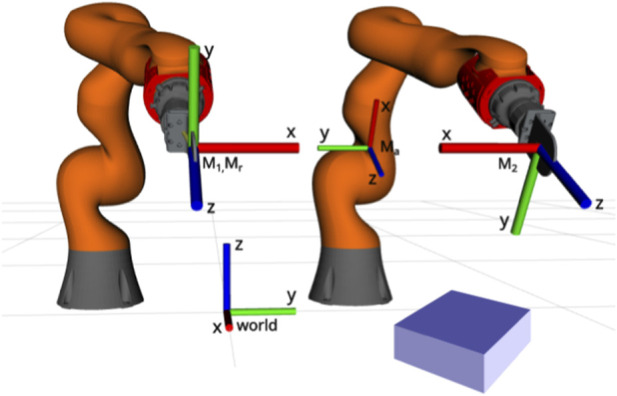
Dual arm setup. Two Kuka iiwa robots with a plate gripper attached to its end-effectors are used to grasp items on a table. The frames used throughout the paper are highlighted here. Namely, $M_{1}$ and $M_{2}$ (motors for grippers 1 and 2), world frame, and absolute $\left(M_{a}\right)$ and relative motors $\left(M_{r}\right)$ .

In some applications, we may need to control multiple manipulators simultaneously. This is the case, for example, when we want to grasp an object with two end-effectors, requiring the coordination of the motion of two arms. The cooperative dual-task space (CDTS), first proposed by Adorno et al. (2010), is a framework that allows to express the collaborative behaviour of two manipulators. In this framework the authors define two variables: the relative variable represents the pose of the first end-effector w.r.t. the second one, and the absolute variable represents a frame located in the middle of the two end-effectors, w.r.t. world frame, corresponding to half of the transformation from one end-effector to another. In this section, we introduce the CTDS using CGA that was presented in Löw and Calinon (2024a) as an extension of Adorno et al. (2010). We first find the relative motor as

$$M_{r}\left(q_{1},q_{2}\right)={\tilde{M}}_{2}\left(q_{2}\right)M_{1}\left(q_{1}\right),$$ (5)

and similarly, the absolute motor can be found as

$$M_{a}\left(q_{1},q_{2}\right)=M_{2}\left(q_{2}\right)\exp\left(\frac{1}{2}\log\left(M_{r}\left(q_{1},q_{2}\right)\right)\right),$$ (6)

where $q_{1}$ and $q_{2}$ denote the joint configurations of the first and second manipulator, respectively. $M_{1}$ and $M_{2}$ are the corresponding end-effector motors found using the forward kinematics formula from Equation 1. Figure 1 illustrates these variables. We omit the derivation of the corresponding analytic Jacobians $J_{r}^{A}\left(q_{1},q_{2}\right)$ and $J_{a}^{A}\left(q_{1},q_{2}\right)$ for brevity and instead refer readers to Löw and Calinon (2024a) for details.

Our extension of the CDTS presented a geometric primitive, called the cooperative pointpair, that corresponds to both end-effector positions simultaneously. This cooperative pointpair is defined as the outer product of the two end-effector points, i.e.,

$$P_{\mathrm{cdts}}=M_{1}\left(q_{1}\right)e_{0}{\tilde{M}}_{1}\left(q_{1}\right)\wedge M_{2}\left(q_{2}\right)e_{0}{\tilde{M}}_{2}\left(q_{2}\right).$$ (7)

## 3 Methods

Admittance control defines a desired dynamic relationship between the external wrenches applied to a robot and its resulting motion. It allows the robot to move compliantly to the external forces and torques making them safer for human-robot interaction and tasks subjected to contact. Moreover, it allows the robots to adapt to unknown environments.

Given a desired motor $M_{d}$ and a current motor $M_{q}$ , the desired apparent impedance of the system can be imposed on the displacement between $M_{d}$ and $M_{q}$ Caccavale et al. (1999). Therefore, we propose an admittance control law formulated using bivector $B_{e}$ , which is the logarithm of the motor error $M_{e}={\tilde{M}}_{q}M_{d}$ .

### 3.1 Admittance control

To impose a desired apparent impedance on the system, we can formulate the dynamics in bivector space as a mass-spring-damper system where the bivectors $B_{e}$ , ${\dot{B}}_{e}$ and ${\ddot{B}}_{e}$ describe the error dynamics in bivector space

$$I\left[{\ddot{B}}_{e}\right]+D\left[{\dot{B}}_{e}\right]+K\left[B_{e}\right]=W_{\mathrm{ext}}-W_{d}.$$ (8)

The tensors I , D and K are inertia, damping and stiffness, respectively. The definition of these tensors can be found in Equation A1 in the Appendix 1. The bivectors $B_{e}$ , ${\dot{B}}_{e}$ , ${\ddot{B}}_{e}$ describe the error dynamics in bivector space. We assume a static target throughout this work, i.e., the desired velocity ${\dot{B}}_{d}=0$ and acceleration ${\ddot{B}}_{d}=0$ . We note that in the general case of a moving target, the velocity error would require an adjoint mapping to transport the desired twist to the current frame before subtraction, as is standard in Lie group formulations of impedance control Seo et al. (2024a), Seo et al. (2024b). Under the static target assumption, however, this adjoint term vanishes, and the bivector velocity error simplifies to ${\dot{B}}_{e}=2\left({\dot{B}}_{d}-{\dot{B}}_{q}\right)=-2{\dot{B}}_{q}$ . We can then use Equation 4 to replace ${\dot{B}}_{e}$ and ${\ddot{B}}_{e}$ using the current twist V and twist acceleration $\dot{V}$ .

Since admittance is dual to impedance, instead of solving for the wrench, we solve for the twist acceleration

$$\dot{V}=I^{-1}\left[W_{\mathrm{ext}}-W_{d}-D\left[V\right]-K\left[B_{e}\right]\right].$$ (9)

We can then find the joint accelerations from the twist acceleration using

$$\ddot{q}=J^{G,-1}\left(\dot{V}-{\dot{J}}^{G}\dot{q}\right),$$ (10)

where $J^{G}$ and ${\dot{J}}^{G}$ are the geometric Jacobian and its time derivative. Note that, this control law is defined here without an explicit reference frame. In practice, a common choice is either the end-effector frame of the manipulator or target frame.

For position or velocity control, the desired joint accelerations ${\ddot{q}}_{d}$ can be integrated once or twice from the current robot state $\left(q,\dot{q}\right)$ using an appropriate time step Δt to either get the joint velocity $\dot{q}$ or position q . If the manipulator that is being controlled allows torque control, the desired torque command $T_{d}$ can be computed using inverse dynamics

$$T=f^{-1}\left(q,\dot{q},\ddot{q}\right).$$

### 3.2 Single arm

In a first step, we demonstrate how the admittance control law from the previous section can be used with a single arm manipulator. Given a desired target motor $M_{d}$ and the current end-effector motor $M_{q}$ , we can find the error bivector as

$$B_{e}=-\log\left({\tilde{M}}_{q}M_{d}\right).$$ (11)

Here, the error bivector $B_{e}$ is expressed w.r.t. the current end-effector frame $M_{q}$ . This formulation is left-invariant on the motor group: the error is always measured in the body frame of the current motor $M_{q}$ , ensuring geometric consistency regardless of the global pose. The twist that is required for the damping term then becomes the end-effector twist $V_{q,\dot{q}}$ and can be computed either from Equation 3 or Equation 4.

### 3.3 Dual arm

The CGA formulation of the cooperative dual task space uses two motors to describe the relationship of the two end-effectors, the relative motor $M_{r}$ and the absolute one $M_{a}$ . Based on these two motors, it is straightforward to define desired motors and obtain the respective error bivectors, $B_{e,r}$ and $B_{e,a}$ using Equation 11. When controlling both absolute and relative motors at the same time, Equation 9 can be extended such that we have individual expressions for the absolute components and the relative ones.

Using the admittance control law in the CDTS then requires the derivation of the geometric Jacobians of the relative and absolute motors. Using the relationship between the geometric and analytic Jacobians from Equation 2, we find them as

$$J_{r}^{G}=-2{\tilde{M}}_{r}J_{r}^{A},$$

and

$$J_{a}^{G}=-2{\tilde{M}}_{a}J_{a}^{A},$$

respectively. Here, $J_{r}^{A}$ and $J_{a}^{A}$ are the analytic Jacobians of the CDTS that we derived in Löw and Calinon (2024a). Consequently, the absolute and relative twists can be found as

$$V_{r}=J_{r}^{G}\dot{q}\;\text{and}\;V_{a}=J_{a}^{G}\dot{q},$$

respectively, where $\dot{q}=\left[{\dot{q}}_{1}\;{\dot{q}}_{2}\right]$ .

Accounting for the two wrenches in the CDTS requires them to be correctly mapped to the relative and absolute frames. For the relative motor, we can find the relative wrench as

$$W_{r}=\frac{1}{2}{\tilde{M}}_{2}\left(W_{2}-W_{1}\right)M_{2},$$ (12)

where $M_{2}$ is the motor of the second arm w.r.t. the world frame, and $W_{1}$ and $W_{2}$ are the external wrenches acting on the end-effectors of robots 1 and 2, respectively, also w.r.t. the world coordinate system. We use $M_{2}$ here as the relative motor $M_{r}$ in Equation 5 is defined w.r.t. the second arm. The absolute wrench can be found as

$$W_{a}={\tilde{M}}_{a}\left(W_{1}+W_{2}\right)M_{a},$$ (13)

where $M_{a}$ is the absolute motor defined in Equation 6.

### 3.4 Practical considerations

#### 3.4.1 Dissipative term

When the robot is redundant with respect to the task, the joint velocities can be different from zero even if the system is in equilibrium. In this case, a dissipative term can be used to prevent the robot from moving after reaching the equilibrium. We opted to add a damping term to the joint velocity in the null space:

$${\dot{q}}_{\text{diss}}=\dot{q}+\left(I-J^{A,-1}J^{A}\right)\left(-k_{\text{diss}}\dot{q}\right).$$

where I is the identity matrix, $k_{\text{diss}}\in \left(0,\infty \right)$ and $\dot{q}$ is the joint velocity obtained by integrating Equation 10.

#### 3.4.2 Unwinding phenomenon

Since motors are an equivalent representation to dual quaternions and also double cover SE(3) , they also suffer from what is known as the unwinding phenomenon. This is a topological issue that is caused by the fact that the motors M and −M represent the same transformation, and it prevents global asymptotic stability, since there are two stable equilibria. In practice, this will cause unnecessary motion when the current end-effector motor $M_{q}$ is topologically closer to $-M_{d}$ rather than $M_{d}$ . There exist, however, already numerous solutions to address this problem for dual quaternions Fonseca et al. (2020) and they can be seamlessly adapted for motors in our proposed CGA admittance control scheme. One possible solution is to check the condition $\|M-1{\|}_{2}\leq \|M+1{\|}_{2}$ and then choose M or −M such that it yields the same result as $M_{q}$ . For instance, we use the following unwinding logic in calculating a bivector $B_{e}$ from a given motor $M_{e}={\tilde{M}}_{q}M_{d}$ :

$$B_{e}=\left\{\begin{array}{ccc} & -\log M_{e}, & if\;\|M_{e}-1{\|}_{2}\leq \|M_{e}+1{\|}_{2},\\ & -\log\left(-M_{e}\right), & otherwise. \end{array}\right.$$

### 3.5 Geometric primitives

One advantage that geometric algebra offers over other frameworks is the direct representation of geometric primitives within the algebra. These primitives can therefore be used in a uniform manner in order to generate the desired behaviors by deriving error bivectors based on their difference. In general, this is achieved by the formula

$$B_{e}=\log\left(M_{e}\right)=\log\left(\frac{1}{c}\left(1+X_{2}X_{1}\right)\right),$$ (14)

where $X_{1}$ and $X_{2}$ are two geometric primitives and c is a normalization constant that depends on the geometric primitives. More details can be found in Lasenby et al. (2019). The resulting motor $M_{e}$ then represents the transformation that transforms $X_{1}$ to $X_{2}$ . In Bilaloglu et al. (2025), this was used to align the end-effector z -axis with the surface normals. Here, we show that other geometric primitives are possible as well to be included in the admittance control law. The general idea is that $X_{1}$ is a geometric primitive that is attached to the robotic system. In that case, $X_{2}$ is a desired geometric primitive on the object where we want to have forceful interactions. By construction, the controller will be stiff when moving away from the geometric primitives and compliant when moving on them.

These primitives can then be used within the admittance controller formulation. To this end, we first define a reference primitive either for the relative or the absolute motor $X_{\mathrm{ref}}$ . Now, using Equation 14, we can find the bivector command that can be used in the admittance controller in Equation 8. For example, if we use a line through the z -axis as the reference, we can transform it to the absolute frame as

$$L_{\mathrm{ref,a}}=M_{a}L_{\mathrm{ref}}{\tilde{M}}_{a}.$$

Then, by defining a target line $L_{\mathrm{target}}$ , we can find motor $M_{e}$ that fulfills $L_{\mathrm{target}}=M_{e}L_{\mathrm{ref,a}}{\tilde{M}}_{e}$ . Using its bivector $B_{e}$ in the controller will then drive the line in the absolute frame towards the target. This control formulation allows completely compliant rotation around the line and translation along it.

## 4 Experimental results

To evaluate our proposed controller, experiments were run on a robotic system with two KUKA LBR iiwa 14 R820 robot manipulators equipped with an ATI Gamma Force/Torque sensor at their wrists, and a plate grippers attached after the sensors, as illustrated in Figure 1. The setup is connected to a computer running Ubuntu 22.04, with 64 GB of RAM and an Nvidia RTX 2080 TI. The controller is implemented in C++. Since we use position control, the control input found in Equation 10 is numerically integrated twice, using Newton’s first-order approximation, to obtain the joint velocity $\dot{q}$ and position q . To prevent reaching the joints’ maximum velocities, they were saturated at 0.2 rad/s. The experiments were run with a sampling time of 1 m. In order to show the performance of the controller, we present two sets of experiments: single-arm and dual-arm experiments.

### 4.1 Single arm experiments

Two types of experiments were performed with the single arm: *hold pose* and *push* experiments. For all results reported in this article using single arm robot, the control law defined in Equation 9 was used with inertia, damping and stiffness tensors chosen as $I=1.5I_{6\text{x}6}$ , $D=300I_{6\text{x}6}$ , $K=80I_{6\text{x}6}$ (same as the parameters used in Fonseca et al. (2020)), where $I_{6\text{x}6}$ is a 6x6 identity matrix.

#### 4.1.1 Hold Pose Experiment

Aiming to demonstrate the behavior of the robot under external wrenches and also in free motion, we proposed the *Hold Pose Experiment*, as explained below.

Consider a scenario where the current motor $M_{q}$ at the beginning of the experiment is equal to the desired one $M_{d}$ . In the absence of an external wrench acting on the robot end-effector, the robot should remain in the desired pose. When an external wrench is applied, the robot should react to it, making $M_{q}$ differ from $M_{d}$ to impose the desired apparent impedance. After releasing the contact, the contact wrench vanishes and the end-effector should return to its original (and desired) pose. This experiment shows the performance of the controller in a single-arm scenario for both contact rich and free-motion tasks.

**FIGURE 2:**
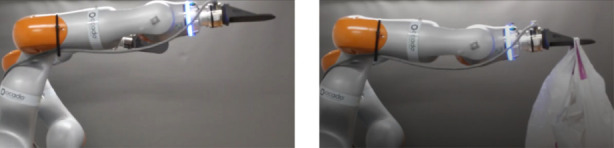
*Left:* the start configuration of the robot, showing the desired pose of the plate gripper. *Right:* the result of the movement after attaching the item with 0.633 kg to the gripper.

**FIGURE 3:**
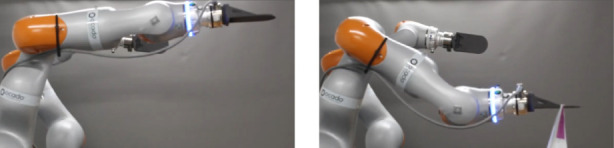
*Left:* the start configuration of the robot, showing the desired pose of the plate gripper. *Right:* the result of the movement after attaching the item with 4.6 kg to the gripper.

We performed this experiment by setting the robot’s end-effector in a horizontal configuration and attaching items of different weights to its plate grippers. The world and gripper frames are shown in Figure 1 and for single arm experiments, the left arm was used. The experiments are shown in Figures 2, 3, where the first item is a plastic model of an Organic Free Range Whole Chicken item whose weight is 0.633 kg and the second one is a Fairy Non Bio Washing Powder for Sensitive Skin 75 Washes item that weighs 4.6 kg. Since the second item is heavier than the first one, the wrench acting on the end-effector is higher, which leads to a higher difference between the desired pose and the actual one.

**FIGURE 4:**
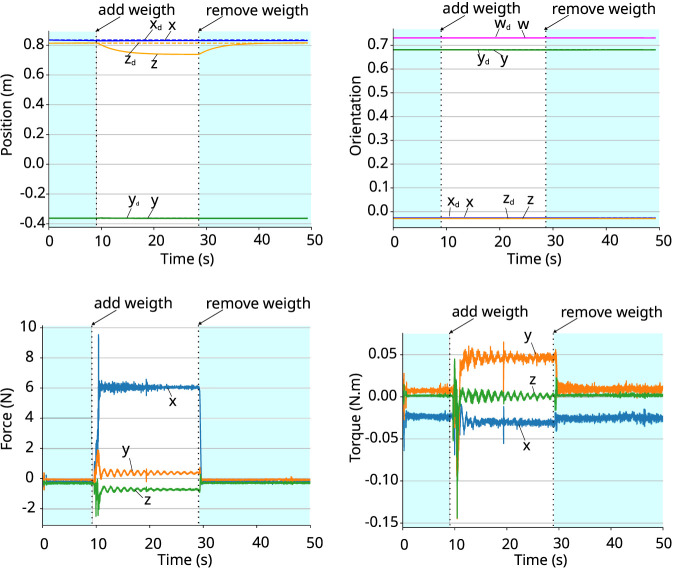
Holding an object that weighs 0.633 kg. *Top-left:* coefficients of the position vector. *Top-right:* coefficients of the quaternion representing the orientation. *Bottom-left:* force. *Bottom-right:* torque.

**FIGURE 5:**
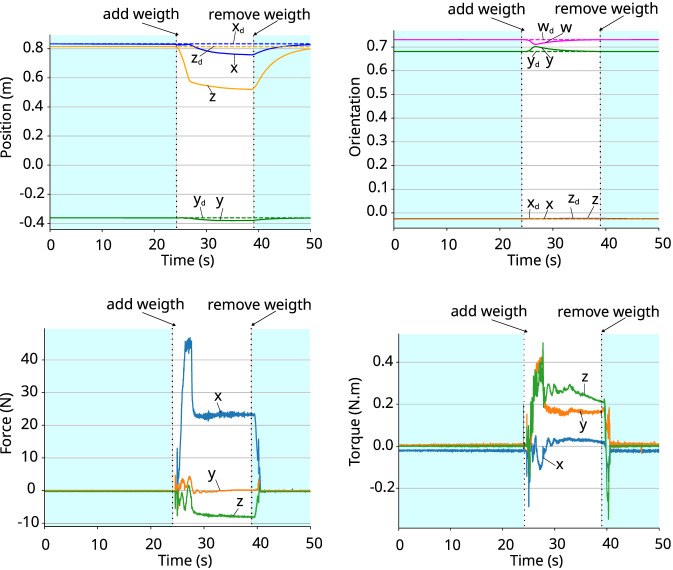
Holding an object that weighs 4.6 kg. *Top-left:* coefficients of the position vector. *Top-right:* coefficients of the quaternion representing the orientation. *Bottom-left:* force. *Bottom-right:* torque.

The coefficients of the poses w.r.t. world frame and the corresponding wrenches acting on the gripper w.r.t. the gripper frame are shown in Figures 4, 5. In both cases, the wrench is close to zero at the beginning of the experiment, when the robot is in free motion, and the gripper pose is equal to the desired one. After adding the object, the external wrench acting on the gripper increases due to the weight of the object, and the robot starts to move compliantly to ensure the desired apparent impedance of the system. When the object is removed, the wrench returns to close to zero and the end-effector converges back to the desired pose. The moments when the weight is added and removed from the gripper are shown in the graphs. This experiment shows that the controller adapts the robot’s motion to ensure the desired apparent impedance according to the wrench acting on it.

#### 4.1.2 Push experiment

**FIGURE 6:**
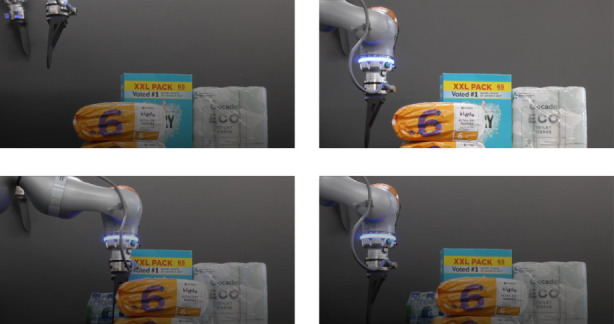
Top left image shows the initial state of the experiment. Top right illustrates the plate gripper pose after going down. Bottom left shows the end of the pushing movement, where the gripper is in contact with the object. Bottom right shows the robot after coming back to the previous pose.

The previous experiment showed that the controller leads to a compliant behavior under external wrenches, when the system is already at the desired pose. In this push experiment, the robot actively moves to push two Kiddo Ultra-Dry Nappies Size 6 (13–18 kg) with 1.119 kg each, totalling 2.238 kg, as shown in Figure 6. This is a common task in industrial applications, where the robot is used to move objects around to create space between items so the arms can pick them up next.

**FIGURE 7:**
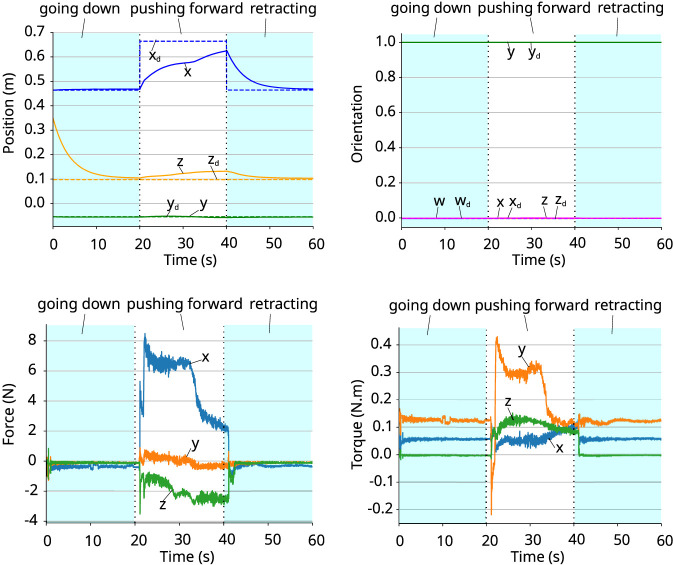
Pushing an object weighing 2.238 kg. *Top-left:* coefficients of the position vector. *Top-right:* coefficients of the quaternion representing the orientation. *Bottom-left:* force. *Bottom-right:* torque.

The experiment has three phases: *go downwards*, *push forward*, and *retract*. Only in the second phase, the robot is in contact with the object and experiences an external wrench. Figure 7 shows the current and desired poses for the experiment and the corresponding wrenches. The black vertical lines in the graphs indicate the phase changes. The first part of the graphs represents the movement of the gripper going down, without any contact with the environment. In this phase, it is possible to see that the wrench values are close to zero, and the orientation of the gripper remains the same during the whole movement. The position in z-axis gets close to the desired one, indicating that the robot is moving as expected. In the second phase, the gripper is moving forward, in the x-axis, and it is in contact with the object it is pushing. The torque values are still small, and not enough to change the orientation of the gripper. The force values are higher, especially in the x-axis. In this phase, the x-axis coefficient of the position changes to try to reach the desired value. Due to the force produced by the contact with the object, the robot is compliant and cannot fully achieve the desired value, although it gets close to it. Also, the z-axis diverges a couple of centimeters from the desired pose, due to the force in the z-axis. In the third phase, when the plate releases the contact with the object and returns to the previous pose, no external force is acting on the plate gripper anymore, and both position coefficients for x and z-axes reaches to the desired values.

### 4.2 Dual arm experiments

**FIGURE 8:**
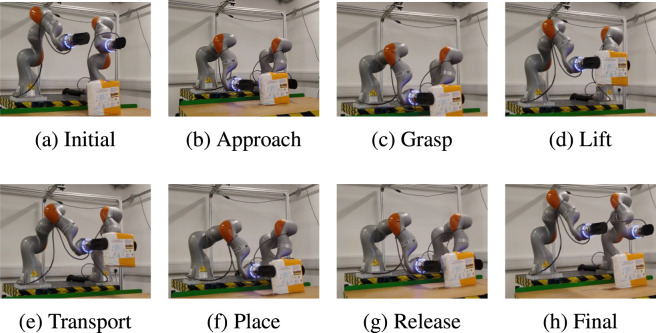
Setup of the dual arm experiments. From left to right, the arm approaches the object, grasps it, lifts it, transports it, places it, and then releases it. **(a)** initial phase, **(b)** approach phase, **(c)** grasp phase, **(d)** lift phase, **(e)** transport phase, **(f)** place phase, **(g)** release phase, **(h)** final phase.

Another task that is common in industrial applications is the cooperative manipulation of an object by two arms. was conducted using objects with varying sizes, weights, and deformability. Here, we report a representative experiment to evaluate the proposed controller in a dual-arm scenario, where both arms jointly grasp and transport an object. The task is broken into different phases, as illustrated in Figure 8. The inertia and damping matrices were chosen empirically as $I=1.5I_{6\text{x}6}$ , $D=300I_{6\text{x}6}$ , respectively, for both absolute and relative poses. The relative stiffness was set as $K=100I_{6\text{x}6}$ , and the absolute as $K=200I_{6\text{x}6}$ . These values were initialized from Fonseca et al. (2020) and further tuned to reduce oscillation when the arm is picking the item that is in contact with a surface, and to ensure stable yet compliant grasping, preventing damages to the item being grasped. Stiffness values were adjusted according to object rigidity. The parameters reported here correspond to those yielding the best performance for the selected object, an Ocado Kiddo Ultra-Dry Nappies Size 6 (13–18 kg) that weighs 1.119 kg.

The task is broken into different phases, as in Figure 8:

- Initial phase: The robot goes to an initial state, defined by an absolute and a relative pose.
- Approach phase: The desired absolute pose is modified, such that the grippers go down, approaching the item. The relative pose remains the same.
- Grasping phase: In order to grasp the item, the relative pose is changed, bringing grippers closer to each other. The absolute pose remains the same.
- Lifting phase: The absolute pose is modified to lift the item, while the relative one is kept unchanged.
- Transport phase: The robot moves the item sideways, which is achieved by a modification of the absolute pose.
- Placing phase: The absolute pose is changed in order for the grippers to go down.
- Releasing phase: The relative pose is changed, which increases the distance between the grippers to release the item. The absolute pose remains unchanged.
- Final phase: The robots lift their grippers by changing the absolute pose to re-initialise the process.

In the *grasping phase*, desired forces of 5 N in the x-axis of each grippers’ frames are chosen in order to ensure a stable grasp. These desired forces are maintained until the *releasing phase*, when it is reduced to 0 N. The desired absolute and relative wrenches $W_{d}$ from Equation 9 are defined according to Equations 13 and 12, respectively.

**FIGURE 9:**
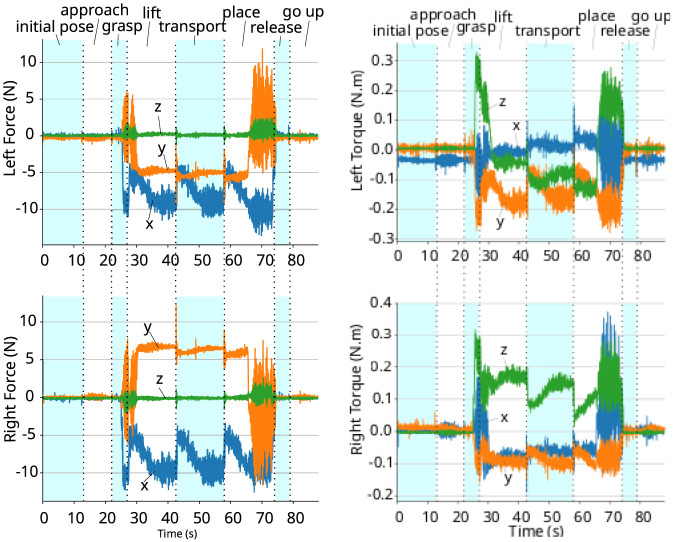
External wrenches applied at each plate gripper, w.r.t. the grippers’ frames. Left: coefficients of the force. Right: coefficients of the torque.

**FIGURE 10:**
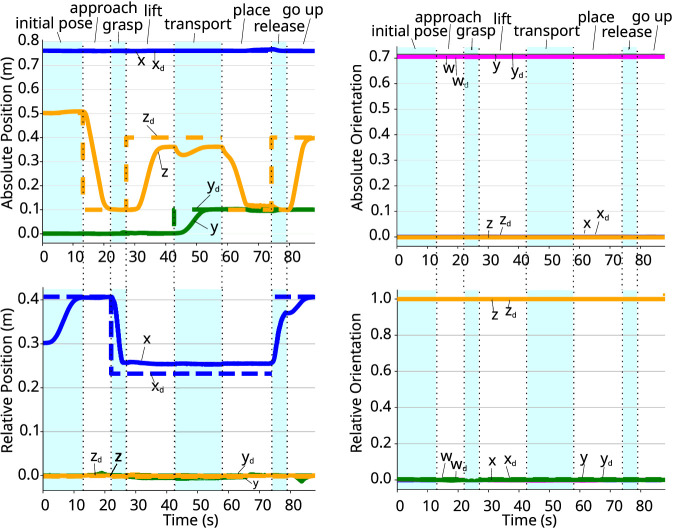
Absolute and relative poses. *Left:* coefficients of the current and desired absolute (top) and relative (bottom) positions. *Right:* coefficients of the quaternion representing the current and desired absolute (top) and relative (bottom) orientation.

Figure 9 shows the compensated external wrenches acting on both arm, while Figure 10 shows the absolute and relative poses. In both the *initial phase* and the *approach phase*, there is no wrench acting on the arms to deviate the poses from the desired ones. When the arms approach the object, in the *grasping phase*, an internal wrench appears on the object when the grippers make contact. Therefore, the controller imposes the desired apparent impedance to the system, preventing the arms to fully reach the desired pose. This can be observed in Figure 10, where the x-axis of the relative position does not reach its target. This error persists throughout all phases where the arms are grasping the object.

In the *lifting phase*, the z-axis of the absolute pose gets closer to its desired value, but does not fully reach it. This is due to the wrench acting on the arms due to gravity. As shown in Figure 9, the y-axis force in each arm is approximately 5 N.^1^ In the *transport phase*, the arms move sideways, and the there is no wrench affecting the movement in the y-axis, allowing y-component to reach its desired value.

In the *placing phase*, the arms lower to place the object on a table. Once the item is released in the *releasing phase*, no wrench acts on them, and all components of the absolute pose achieve the desired values. The same holds true for the *final phase*.

Notably, there is a temporary error in the x-axis of the relative position during the *releasing phase*. This occurs because a set timeout was reached before the pose was fully attained. However, this error corrects itself in the subsequent phase as the movement continues. Throughout the entire experiment, the orientation of the grippers remains practically unchanged, indicating that the torque components observed in both arms were very small (less than 0.4 N).

### 4.3 Dual arm line experiments

Traditional bi-manual control strategies often enforce a full 6-DoF (Degrees of Freedom) absolute pose constraint, rigidly defining both the position and orientation of the manipulated object. However, in many real-world logistics applications such rigid constraints are often unnecessary and potentially detrimental to task success. Instead, we implement a task-space constraint strategy that controls only the line of action (specifically the z -axis of the absolute pose). By prioritizing axial alignment while relaxing constraints on the “roll” angle and translation along the vector, this approach effectively reduces the task requirement from 6-DoF to 4-DoF. This reduction affords the system to execute other tasks in the null-space of the primary task, enhancing overall performance and adaptability. It also introduces mechanical compliance, allowing the arms to adapt to uncertainties in object geometry and environmental interactions. To validate this approach, we conducted an experiment where the dual-arm system is tasked with lifting and transporting an object using only the line of action constraint. The experimental setup mirrors that of the previous dual-arm experiments, with the same KUKA iiwa manipulators and plate grippers. The object is initially placed on a table, and the arms are commanded to grasp it from either end, lift it, and transport it to a designated location. In this experiment, we are controlling the absolute line corresponding to the z -axis of the desired absolute pose, while leaving the other components free to adapt. The relative pose is fully constrained to maintain a stable grasp.

Here, the inertia and damping matrices were chosen empirically as $I=1.5I_{6\text{x}6}$ , $D=600I_{6\text{x}6}$ , respectively, for both absolute and relative variables. The relative stiffness was set as $K=100I_{6\text{x}6}$ , and the absolute as $K=200I_{6\text{x}6}$ . The controller gains were chosen to have a compliance in the system while achieving the desired pose/line in the absence of contact wrenches. The desired forces of 5 N in the x-axis of each grippers’ frames are also chosen in order to ensure a stable grasp, similar to the previous dual-arm experiment.

**FIGURE 11:**
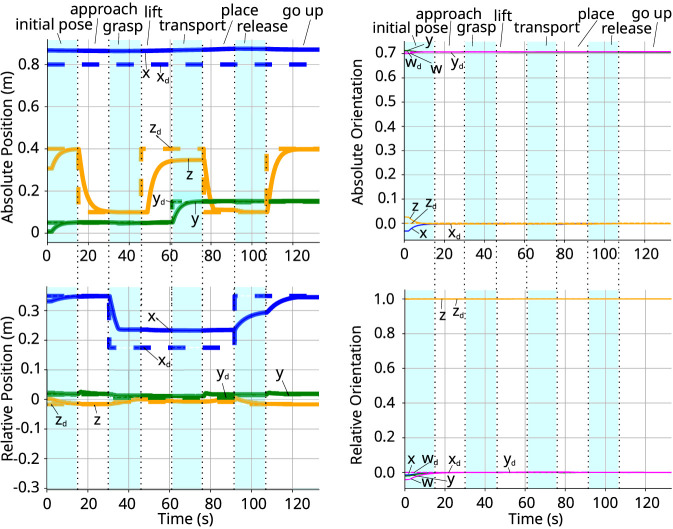
Absolute and relative poses for the dual-arm line controller. *Left:* coefficients of the current and desired absolute (top) and relative (bottom) positions. *Right:* coefficients of the quaternion representing the current and desired absolute (top) and relative (bottom) orientation.

**FIGURE 12:**
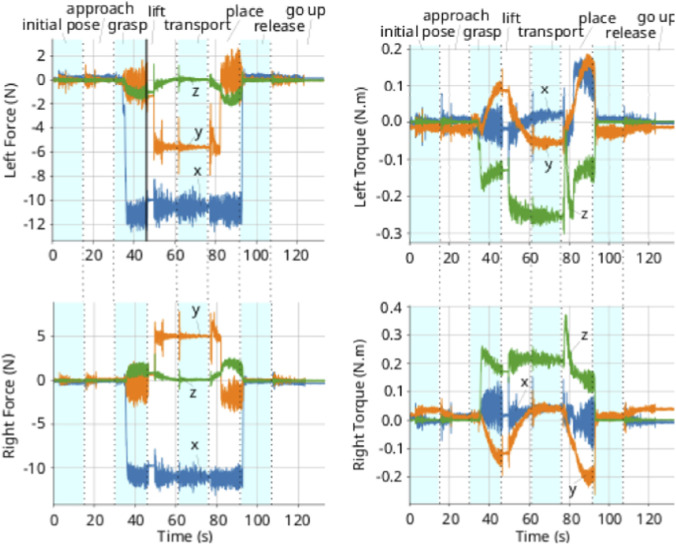
External wrenches applied at each plate gripper, w.r.t. the grippers’ frames when executing the dual-arm line controller. *Left:*coefficients of the force. *Right:*coefficients of the torque.

The compensated external wrenches exerted on the manipulators are illustrated in Figure 12, while the resulting trajectory for both absolute and relative coordinates is depicted in Figure 11. Although we only control the line represented by the z-axis of the absolute pose, all components of both absolute and relative positions and orientations are plotted for comprehensive analysis. Also, since the absolute pose is ploted w.r.t. the world frame, the z-axis corresponds to the line pointing to the front of the robot, similar to Figure 1, which in this case is equivalent to the x-axis of the world frame.

During the *initial* and *approach phases*, the system moves in free space without external disturbances, allowing the poses to track the reference trajectories closely. A deviation is observed in the x-axis of the absolute pose. Unlike the previous experiment, this variable does not converge to the reference value. This behavior is by design: since the control law restricts only the line of action, the rotational orientation around that line is left unconstrained, as well as the point in the line, allowing the arms to find a natural equilibrium without fighting to enforce a specific angle or position.

Upon entering the *grasping phase*, the contact between the grippers and the object generates a significant internal wrench. In response, the admittance controller prevents the end-effectors from strictly enforcing the target relative position. This is evident in Figure 11, where the x-component of the relative position maintains a steady-state offset to sustain the grasp force. Transitioning to the *lifting phase*, the absolute z-position approaches the setpoint but exhibits a minor deviation caused by the gravitational load. As detailed in Figure 12, the vertical force component distributed to each arm is approximately 5N, similar to what is observed in the dual arm experiment in the previous section, which corresponds to the weight of the item being manipulated.

During the *transport phase*, there is only a lateral motion (y-axis) and no wrench is acting on this axis, enabling that specific component to converge fully to its target. Finally, the arms descend during the *placing phase* to deposit the item. Upon execution of the *releasing phase*, the contact forces vanish. In the *final phase*, the positional variables and the relative pose return to their desired values; however, the absolute x-axis position remains at the point determined by the grasp dynamics, confirming that the line-control strategy successfully decoupled the components from the line.

**FIGURE 13:**
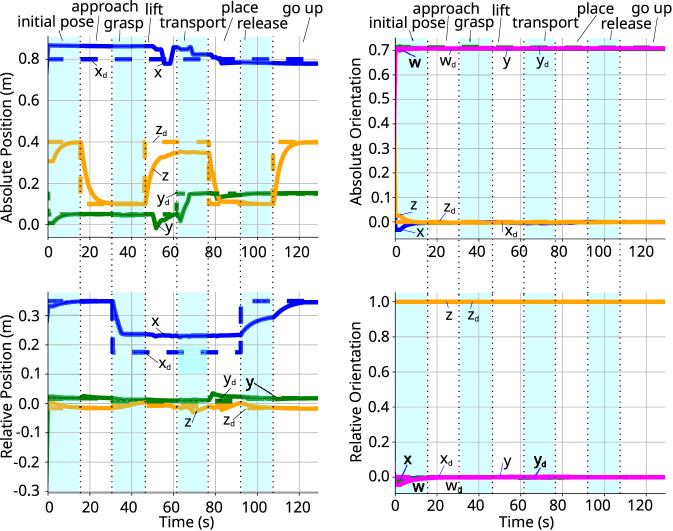
Absolute and relative poses for the dual-arm line controller, with human interaction. *Left:* coefficients of the current and desired absolute (top) and relative (bottom) positions. *Right:* coefficients of the quaternion representing the current and desired absolute (top) and relative (bottom) orientation.

**FIGURE 14:**
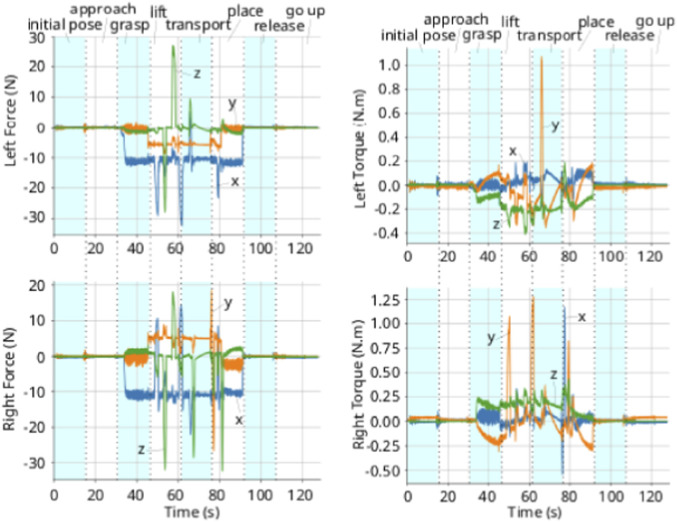
External wrenches applied at each plate gripper, w.r.t. the grippers’ frames when executing the dual-arm line controller, with human interaction. *Left:* coefficients of the force. *Right:* coefficients of the torque.

The same experiment was repeated with human interaction, where a person applied forces to the gripper while the robot was lifting and transporting the object. The results, shown in Figures 13, 14, are similar to those presented above, with the addition of disturbances in the wrenches and poses due to human interaction. The controller successfully maintained the line of action while adapting to the external forces applied by the human, demonstrating its robustness and compliance in dynamic environments. This experiment highlights the effectiveness of the line-control strategy in facilitating cooperative manipulation tasks while allowing for adaptability and safety in human-robot interaction scenarios. The variations in the x-axis of the absolute position during human interaction are attributed to the relaxed constraints of the line-control approach, allowing the system to naturally adjust to external disturbances without rigidly enforcing a specific position along the line.

## 5 Discussion

### 5.1 Comparison to baseline methods

The proposed controller was not compared to other benchmark methods in the experiments. We acknowledge this as a limitation. The most natural baseline is a dual quaternion admittance controller Fonseca et al. (2020); Fonseca (2021), which shares the same algebraic structure as the CGA formulation, i.e., the motor group is isomorphic to the unit dual quaternion group. For pose-tracking tasks, the two formulations are numerically equivalent by construction, making a quantitative performance comparison uninformative. The novel contribution of the geometric primitive controller (Section 3.5) has no direct counterpart in the existing dual-arm admittance control literature, as prior formulations operate on poses rather than geometric primitives. Future work should include a systematic comparison against SE(3)-based geometric impedance controllers Seo et al. (2024a), Seo et al. (2024b) to quantify any practical differences in convergence, compliance behaviour, and robustness.

### 5.2 Energy

We derive the admittance control law in CGA from the Hamiltonian of the system in bivector space, similar to the dual quaternion equivalent that was presented in Fonseca et al. (2020). The Hamiltonian can be found as

$$e=k+u=-\frac{1}{2}V\cdot I\left[V\right]-B\cdot K\left[B\right],$$

where k and u are the kinetic and potential energy. I and K denote the inertia and stiffness tensors that are defined in the way we presented in. The time derivative of the Hamiltonian can then be found as

$$\dot{e}=-V\cdot I\left[\dot{V}\right]-2B\cdot K\left[\dot{B}\right],$$

The standard methods for ensuring passivity, such as energy tanks, can be applied in this framework as well. We will not go into further detail on this topic, since the focus of this article lies in demonstrating how geometric algebra can be used to simplify the geometric modeling for admittance control tasks, expand across different tasks.

## 6 Conclusion

In this work, we presented a dual-arm admittance control framework leveraging CGA to enhance the manipulation capabilities of robotic systems. By utilizing CGA, we achieved a compact and intuitive representation of spatial transformations, enabling efficient computation and improved task modeling.

The proposed approach was validated through experiments, demonstrating its effectiveness in handling single and dual-arm coordination tasks. In the absence of external wrenches, the system successfully reached the desired poses, while in the presence of external wrenches, it adapted its motion to maintain the desired impedance. The results indicate that the CGA-based admittance control framework is capable of providing compliant and coordinated manipulation in single-arm and dual-arm robotic systems.

Future work will focus on extending this framework to multi-arms/fingers and incorporating adaptive stiffness term so the controller can automatically adapt to different manipulated objects, with various weights and deformability. Furthermore, we will expand the controller to account for inaccuracies in both perception and the robot itself, such as tendon driven robots. Moreover, CGA can also be used to model multiple contact points, when using tactile sensors Giovinazzo et al. (2024).

## Data Availability

The original contributions presented in the study are included in the article/supplementary material, further inquiries can be directed to the corresponding author.

## Footnotes

1 According to Equation 13, the absolute wrench (or external wrench) is the sum of the wrenches in each arm, transformed to be with respect to the absolute frame. Since the weight of the object is 1.119 Kg, the absolute wrench is around 10.96N, which if divided equally between the arms, results in ∼ 5.48 N on each arm.

## Appendix A

### Tensor

Given a matrix $A=diag\left(A_{\phi },A_{p}\right)$ , where $A_{\phi }$ and $A_{p}$ are the rotation and translation terms, the tensor A can be constructed similar to Löw and Calinon (2024b) as

$$\begin{array}{cc} A_{\phi }^{e_{23}} & =a_{\phi_{xx}}e_{23}-a_{\phi_{xy}}e_{13}+a_{\phi_{xz}}e_{12},\\ A_{\phi }^{e_{13}} & =-a_{\phi_{xy}}e_{23}+a_{\phi_{yy}}e_{13}-a_{\phi_{yz}}e_{12},\\ A_{\phi }^{e_{12}} & =a_{\phi_{xz}}e_{23}-a_{\phi_{yz}}e_{13}+a_{\phi_{zz}}e_{12},\\ A_{p}^{e_{01}} & =a_{p_{xx}}e_{01}+a_{p_{xy}}e_{02}+a_{p_{xz}}e_{03},\\ A_{p}^{e_{02}} & =a_{p_{xy}}e_{01}+a_{p_{yy}}e_{02}+a_{p_{yz}}e_{03},\\ A_{p}^{e_{03}} & =a_{p_{xz}}e_{01}+a_{p_{yz}}e_{02}+a_{p_{zz}}e_{03}. \end{array}$$
(A1)
